# Lung cancer screening completion among patients using decision aids: a systematic review and meta-analysis

**DOI:** 10.1007/s10552-025-01987-4

**Published:** 2025-03-18

**Authors:** Alexander Antigua-Made, Sabrina Nguyen, Ali Rashidi, Wen-Pin Chen, Argyrios Ziogas, Gelareh Sadigh

**Affiliations:** 1 Anne Burnett School of Medicine, Texas Christian University, Fort Worth, TX USA; 2 Department of Radiological Sciences, University of California Irvine, 101 The City Dr S, Orange, Irvine, CA 92868 USA; 3 Department of Radiology and Imaging Sciences, Emory University, Atlanta, GA USA; 4 Chao Family Comprehensive Cancer Center, University of California Irvine, Irvine, CA USA; 5 Department of Medicine, Genetic Epidemiology Research Institute, University of California Irvine, Irvine, CA USA

**Keywords:** Lung cancer, Screening, Decision aid, Systematic review, Meta-analysis

## Abstract

**Purpose:**

Utilization of lung cancer screening (LCS) among eligible patients remains low at 16% in 2022. In this systematic review and meta-analysis we assessed the (a) LCS completion rate, and (b) intention to complete LCS, among patients who receive patient decision aids (PDAs).

**Methods:**

PubMed, Cochrane, Scopus, CINAHL, and Web of Science were searched for articles published in English between 1 January 2011, and 28 February 2023. Two independent reviewers selected randomized controlled trials and prospective cohort studies that reported PDA interventions targeting either LCS completion rate or intention to complete LCS. Quality appraisal and data extraction were performed independently by 2 reviewers using the National Heart, Lung, and Blood Institute quality assessment tool. A random-effects model meta-analysis was performed. Reporting followed the Preferred Reporting Items for Systematic Review and Meta-analyses guidelines.

**Results:**

Thirteen studies with 2,277 total participants (51.5% male) were included. The pooled LCS completion rate across all follow-up periods (range, 1–6 months) was 40% (95% confidence interval [CI], 15–65%) with an I^2^ of 97% for heterogeneity. Pooled intention to complete LCS among patients who received PDA across all follow-up periods (same day to 3 months) was 57% (95% CI, 34% to 80%) with significant heterogeneity (I^2^) of 96% (*p* < 0.0001). No publication bias was identified.

**Conclusions:**

LCS completion and intention to complete LCS among patients who use PDAs is high. Our findings support the need to implement PDAs in clinical practice which could further facilitate shared decision-making and improve LCS uptake among eligible patients.

**Supplementary Information:**

The online version contains supplementary material available at 10.1007/s10552-025-01987-4.

## Introduction

Lung cancer is the leading cause of cancer-related death in the United States with a 5 year survival rate of 25.4% [1]. This alarming statistic underscores the importance of annual lung cancer screening (LCS), which has been shown to reduce mortality by 20% [2]. The 2021 US Preventive Services Task Force (USPSTF) recommends annual screening for lung cancer using low-dose computed tomography (LDCT) among individuals aged 50 to 80 years, who have a smoking history of 20 pack-years and are either current smokers or have quit smoking within the past 15 years [3]. Since 2015, LCS for eligible patients following documented shared decision-making in the electronic medical record (EMR) has been covered by both public and most private insurance [4, 5]. However, utilization of LCS remains low at 16% among eligible patients in 2022 [6]. Furthermore, only 9% of potentially-eligible patients discussed the exam with their provider [7].

Despite the 20% reduction in mortality associated with annual LDCT, there are various barriers to LCS. These include patients’ limited awareness of LCS availability, its benefits and insurance coverage, financial concerns about the cost of LDCT or downstream tests following a positive LDCT, societal stigma around smoking, and the fear of receiving a lung cancer diagnosis [8]. Referring providers also face barriers in effective LCS implementation. These include unfamiliarity with or skepticism toward the evidence supporting LCS guidelines, difficulty in identifying eligible patients and facilitating the shared decision-making process, and challenges in managing results from LCS [8].

Implementing interventions such as patient decision aids (PDAs) that outline the risks and benefits of LCS is crucial for overcoming some of these barriers and providing essential support to patients as they decide on LCS [9]. Documentation of shared decision making (SDM) regarding LCS using a decision aid is one of CMS criteria for LDCT coverage [5], yet its utilization remains low [10]. Further, decision aids are designed to enhance decisional quality regardless of decisional outcome. The purpose of this systematic review and meta-analysis was to assess two decisional outcomes among patients receiving PDAs: [1] LCS completion rate and (2) intention to complete LCS.

## Materials and methods

The research methods of this review are reported following the Preferred Reporting Items for Systematic Review and Meta-Analysis (PRISMA) statement [11].

### Data sources and searches

Five electronic databases (MEDLINE (PubMed), Cochrane, Scopus, CINAHL (Cumulative Index to Nursing and Allied Health Literature), and Web of Science) were searched for articles published in English between 1 January 2011, and 28 February 2023, using keywords and MESH terms as outlined in Supplementary Table 1 and 2. All identified studies were imported into Covidence, a web-based collaboration software platform that streamlines the production of systematic and other literature reviews [12].

### Study selection

Eligible studies included prospective pre- and post-intervention designs or randomized control trials (RCTs) reporting interventions involving educational materials or PDA components discussing LCS benefits and risks. They reported at least one of two outcomes: patient completion of LCS or intent to undergo LDCT for LCS. Excluded were review articles, retrospective databases analyses, and studies without published results. Two independent reviewers (AA, SN) initially screened study titles and abstracts, then reviewed full texts to assess eligibility, with conflicts resolved by a third reviewer (GS).

### Data extraction and quality assessment

Data was extracted using a standardized format that included general study information (title, authors, study design), participant demographics, intervention details, and study outcomes such as LCS completion rate, and intent to screen. Data from eligible studies were extracted independently by two reviewers (AA, SN), with conflicts resolved by a third reviewer (GS).

Quality assessment of included studies was independently performed by two reviewers (AA, SN) using the National Heart, Lung, and Blood Institute's (NHLBI) Study Quality Assessment Tools for Controlled Intervention studies and Before-After (Pre-Post) studies [13] with conflicts resolved by a third reviewer (AR). Criteria assessed included selection bias, comparability of study groups, outcome assessment, and follow-up adequacy. The evaluation also addressed blinding of participants and personnel, as well as allocation concealment in RCTs.

### Data synthesis and analysis

A meta-regression was conducted via a random-effects regression model for rates of LCS completion or intent among patients who used PDAs. Post-intervention outcome estimates from the pre-post design studies and outcome estimates in the intervention arms of RCTs were used. Given none of the RCTs had an arm where PDAs were not offered, we could not assess the effectiveness of PDAs' use versus non-use on LCS completion or intent to complete, and thus, we only reported the outcomes among those who used PDAs.

The generalized Q-statistic heterogeneity estimator was utilized, and the sampling weight was used and applied. [14, 15] The adjusted standard errors of the estimated coefficients were calculated. [16–18] A Cochran’s Q test and the I^2^ index were used to estimate the heterogeneity in meta-analyses. A rank correlation test and Egger’s test were performed to examine the publication bias. [19] A forest plot and funnel plot were constructed. The meta-analysis was performed by utilizing the R metafor package2 version 4.2–0. [20].

## Results

**Fig. 1:**
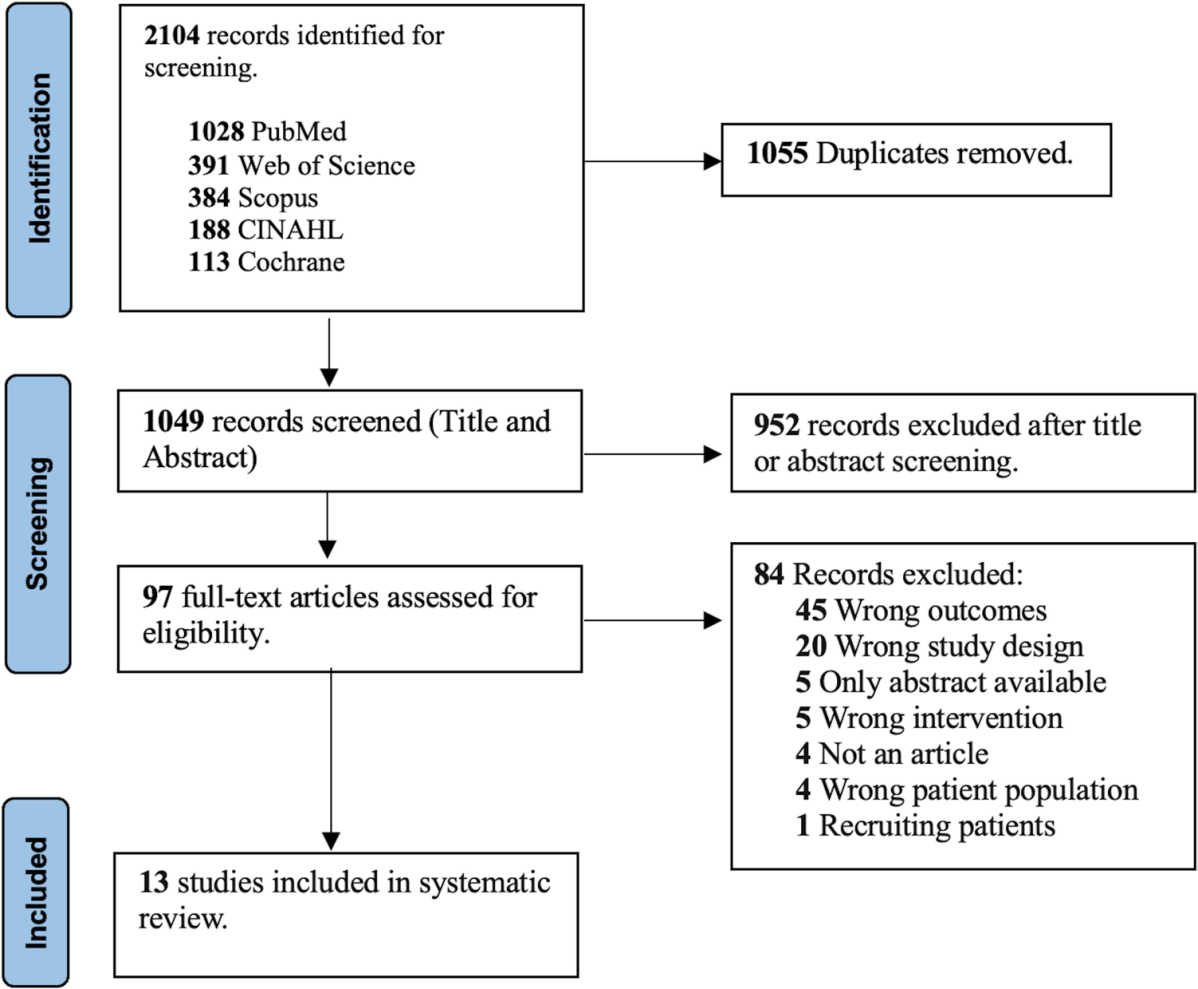
PRISMA Flow Sheet. *CINAHL* Cumulated Index to Nursing and Allied Health Literature. (Color figure online)

Our initial search identified 2,104 studies. After removing duplicates, we screened the title and abstract of 1,049 studies. Excluding those that did not meet our eligibility criteria, we reviewed 97 full-text studies for inclusion and ultimately included thirteen studies with 2,277 total participants [21–33] (Fig. 1).

Nine pre-post intervention design studies [21–23, 26–28, 31–33] and four RCTs [24, 25, 29, 30] were included. One of the RCTs, Kathuria et al. [24] reported results for two pilot RCTs (pilot 1 and 2), resulting in 14 total trials in this study.

In majority of trials LCS eligibility was confirmed prior to delivery of PDAs or educational materials. However, in Williams et al. [26] LCS educational materials were offered to community members regardless of eligibility, and in Dharod et al. [33] the PDA was offered to patients potentially eligible for LCS, but eligibility was confirmed after delivery of PDA.

The mean age of participants across studies ranged between 58 and 66 with 51.5% of participants being male. A total of 52.7% of participants were white, 43.2% were Black and 4.1% were other races. The baseline characteristics of the included studies are shown in Table 1.

**Table 1:** Characteristics of included studies

Author, country, year, reference	Study design	Eligibility criteria	Intervention	Control group	Outcomes	Follow up period	Total *n*	# Intervention	# Control	Mean age	Sex (M/F)	Race
Owens et al., United States, 2023 [21]	Pre-post intervention design	Met the CMS LCS eligibility criteria: (1) age 55–77 years; (2) asymptomatic (no signs or symptoms of lung cancer); (3) tobacco smoking history of at least 20 pack-years; (4) current smoker or former smoker who has quit smoking within the last 15 years; (5) Medicare or Medicaid eligible	PDA “Is lung cancer screening for you?” was adapted for use on mobile and computer-based devices through the Redcap survey platform. A research coordinator provided participants with an overview of the PDA and instructed them to complete the PDA using a tablet computer during a doctor’s appointment After PDA completion, participants followed with an SDM conversation with their provider	NA	LCS completion and intent	same day for intent; 30 days for completion	33	33	33	66.5	12/21	White (*n* = 14) Black (*n* = 18) Other (*n* = 1)
Niranjan et al United States 2023[22]	Pre-post intervention design	Met the USPSTF LCS eligibility criteria: (1) age 55–80 years; (2) 30 pack-year tobacco smoking history; (3) Current smoker or former smoker who quit within the past 15 years	30 min educational session delivered by a community health advisor	NA	LCS completion	NR	100	100	/	62.94	46/54	White (*n* = 3); Black (*n* = 97); Other (*n* = 0)
Fukunaga et al., United States, 2022 [23]	Pre-post intervention design	Met the USPSTF LCS eligibility criteria: (1) age 55–80 years;(2) 30 pack-year tobacco smoking history; (3) current smoker or former smoker who has quit smoking within the last 15 years	Review of a PDA independently, followed by a SDM visit using the DA to explain benefits and harms of LCS	NA	LCS completion		23	23	23	65.8	13/10	NR
Kathuria et al. pilot 1, United States 2022 [24]	Randomized control trial	Hospitalized individuals who met the USPSTF LCS eligibility criteria: (1) age 55–80 years (2) 30-pack years smoking history; (3) currently smoked cigarettes; (4) spoke, read, and understood English; (5) had a PCP in Boston Medical Center or affiliated Community centers; (6) No history of severe comorbidities expected to limit life expectancy or chest CT in the past year; (7) ability to tolerate surgical resection of a lung cancer	PDA “Is Lung Cancer Screening Right for me?” was utilized by a nurse practitioner to facilitate SDM during an inpatient admission and continuing the discussion in the ambulatory setting with an outpatient provider Additionally, patients received tobacco dependence counseling	Handing out DA without reviewing.; Tobacco dependence counseling	LCS completion and intent	3 months	100	52	48	61.8	65/35	White (*n* = 30); Black (*n* = 60); Other (*n* = 10)
Kathuria et al. pilot 2, United States, 2022 [24]	Randomized control trial	Hospitalized individuals who: met the USPSTF LCS eligibility criteria: (1) age 55–80 years (2) 30-pack years smoking history; (3) currently smoked cigarettes; (4) spoke, read, and understood English; (5) had a PCP in Boston Medical Center or affiliated Community centers; (6) No history of severe comorbidities expected to limit life expectancy or chest CT in the past year; (7) ability to tolerate surgical resection of a lung cancer	PDA “Is Lung Cancer Screening Right for me?” was utilized by a nurse practitioner to facilitate SDM during an inpatient admission and continuing the discussion in the ambulatory setting with an outpatient provider Additionally, a navigator assisted in patient navigation to overcome patient barriers to LCS completion including addressing health-related social needs, ordering LCS and assistance with scheduling Patients received tobacco dependence counseling	Handing out DA without reviewing.; Tobacco dependence	LCS completion and intent	3 months	21	10	11	64	15/6	White (*n* = 5); Black (*n* = 14); Other (*n* = 2)
Clark, SD et al. United States, 2022 [25]	Randomized control trial	Met the USPSTF LCS eligibility criteria: (1) age 55–80 years;(2) 30 pack-year tobacco smoking history;(3) current smoker or former smoker who quit within the past 15 years	A 4.5 min video PDA that included information about incidental findings	DA video without information on incidental finding	Intent	Same day	348	173	175	64.5	169/179	White (*n* = 254); Black (*n* = 61); Other (*n* = 33)
Williams et al., United States, 2021 [26]	Pre-post intervention design	(1) English-speaking men and women; (2) Aged 21 to 80 years; (3) able to complete an electronic survey, with or without assistance	Four 90 min sessions, conducted once a week, addressing key concepts from the Health Belief Model In addition, all participants were provided with a card containing a checklist of eligibility criteria for LCS	NA	LCS completion	3 months	481(37 met the eligibility criteria for LCS)	37	37	58.3	351/130	White (*n* = 20); Black (*n* = 444); Other (*n* = 17)
Clark SD et al., United States, 2021 [27]	Pre-Post intervention design	Met the USPSTF LCS eligibility criteria: (1) age 55–80 years;(2) 30 pack-year tobacco smoking history;(3) current smoker or former smoker who quit within the past 15 years	A video-based 3.5 min DA was given to the participants	NA	Intent	Same day	219	219	/	64.7	93/126	White (*n* = 165); Black (*n* = 38); Other (*n* = 16)
Fagan et al United States 2020[28]	Pre-post intervention design	Met the USPSTF LCS eligibility criteria: (1) age 55–80 years; (2) 30 pack-year tobacco smoking history; (3) Current smoker or former smoker who quit within the past 15 years. (4) Has not received a LDCT scan within the last year	Review of educational materials with a decision counselor and a decision counseling session using an online software	NA	LCS completion	90 days	28 (recruited)	20 (underwent DCP intervention)	/	62.4	13/15	White (*n* = 22); Black (*n* = 5); Other (*n* = 1)
Volk et al., United States 2020 [29]	Randomized control trial	Tobacco quit line clients (ages 55–77 years) who reported a 30-plus pack-year smoking history and who spoke English	A 9.5-min narrated PDA video, *Lung Cancer Screening: Is It Right for Me?*	2-page brochure from a lung cancer advocacy group with structured questions about lung cancer screening	LCS completion and intent	1 week for intent; 6 month for completion	516	259	257	NR	196/320	White (*n* = 362); Black (*n* = 138); Asian (*n* = 0); Other (*n* = 16)
Ruparel et al., United Kingdom 2019 [30]	Randomized control trial	(1) Smokers or former smoker (who quit within the past 5 years); (2) age 60–75 years	information film and PDA booklet	DA booklet	LCS completion	NR	229	120	109	66	109/119	White (*n* = 190); Black (*n* = 21); Other (*n* = 11)
Dharod et al., United States 2019[33]	Pre-post intervention design	primary care patients who were potentially eligible for lung cancer screening based on age and smoking history	Web-based decision aid for LCS, mPATH-Lung (mobile patient technology for health—Lung), designed to ensure patients were well informed of risks and benefits prior to in-person shared decision making with a medical provider	NA	LCS completion	4 months	99 eligible for screening	99	/	NR for patients eligible	NR for patients eligible	NR for patients eligible
Hoffman et al., United States, 2018 [31]	Pre-post intervention design	(1) English-speaking men and women; (2) age 55–80 years; (3) current smokers or former smokers who had quit within the past 15 years; (3) no history of lung cancer	PDA “Is lung cancer screening for you?” was adapted for use in video format	NA	Intent	Same day	30	30	30	61.5	15/15	White (*n* = 19); Black (*n* = 0) Other (*n* = 11)
Reuland et al., United States 2018 [32]	Pre-post intervention design	current or former smokers, ages 55–80 years, and eligible for screening based on current screening guidelines	6 min video decision aid	NA	LCS completion and intent	Intent same day; LCS completion 3 months	50	50	/	63	26/24	White (*n* = 29); Black (*n* = 15); Other (*n* = 6)

*PDA* patient decision aid; SDM Shared decision making, *CMS* Centers for Medicaid & Medicare Services, *LHC *lung health check

*LSUT* Lung Screen Uptake Trial, *CHA* community health advisors, *DCP* Decision Counseling Program, *NR* Not reported, *NA* Not applicable

^*^The reported demographic for this study is based on all patients included who may or may not have met the criteria to receive LCS

PDAs and educational materials were used in various formats and delivered differently across the studies (Table 2). In five studies PDAs were offered in videos ranging between 3.5 and 9.5 min [25, 27, 29, 31, 32]. Two studies including the two Kathuria et al. RCTs used paper-based PDAs [23, 24], one study used a web-based PDA [21], one study used a combination of video and paper PDAs [30], one study used a combination of web-based and video PDAs [33], and one study used a combination of web-based and paper PDAs [28]. Format of PDAs or education materials was unknown in two studies [22, 26].

**Table 2:** Format and delivery of PDA and educational materials across studies

Author, country, year, reference	PDA or educational material format				PDA or educational material delivery		Shared decision-making encounter				Ordering of LCS	Assistance with LCS scheduling
	Video	Paper	Web-based	Unknown	Independent review by patients	Delivery by healthcare personnel	No	Yes	Patient instructed to talk to pcp or LCS clinic	PCP was notified of patients’ eligibility		
Owens et al., United States, 2023 [21]			X		X	X (study coordinator)		X (after PDA review)				
Niranjan et al United States 2023[22]				X		X (community health worker)			X			
Fukunaga et al., United States, 2022 [23]		X			X			X (after PDA review)				
Kathuria et al. (pilot 1), United States 2022 [24]		X (both arms)			X (control arm)	X (nurse practitioner in intervention arm)			X (control arm)	X (intervention arm)		
Kathuria et al. (pilot 2), United States, 2022 [24]		X (both arms)			X (control arm)	X (nurse practitioner in intervention arm)		X (during PDA review encounter)	X (control arm)		X (intervention arm)	X (intervention arm)
Clark, SD et al. United States, 2022 [25]	X (both arms)				X (both arms)		X (both arms)					
Williams et al., United States, 2021 [26]				X		X (community health worker)	X					
Clark SD et al., United States, 2021 [27]	X				X		X					
Fagan et al United States 2020[28]		X	X			X (decision counselor)		X (during PDA review encounter)	X (referral to LCS clinic)			
Volk et al., United States 2020 [29]	X (intervention arm)		X (control arm)		X (both arms)		X		X (both arms)			
Ruparel et al., United Kingdom 2019 [30]	X (intervention arm)	X (both arms)			X (both arms)			X (after PDA review)			X	
Dharod et al., United States 2019[33]	X		X		X				X (referral to LCS clinic)			
Hoffman et al., United States, 2018 [31]	X				X		X					
Reuland et al., United States 2018 [32]	X				X		X					

*LCS* lung cancer screening, *PDA* patient decision aid; PCP, primary care provider

None of the RCTs had an arm where no PDA or educational material was offered to participants. Kathuria et al. [24] provided control arm participants from both pilots a paper-based PDA without actively reviewing it. Clark et al. [25] gave control arm participants a video PDA similar to intervention arm, that only omitted information about incidental findings identified during LCS. Volk et al. [29] gave control arm participants a paper brochure containing information about lung cancer screening, and Ruparel et al. [30] gave participants a paper PDA booklet.

In eight studies, including control arm of both Kathuria et al. trials [24], PDAs or educational materials were reviewed by patients independently [23, 25, 27, 29–33], while in one study a research coordinator provided an overview of PDA followed by an independent review by patient [21]. Further, in two studies educational materials were delivered by community health workers [22, 26], and in another two studies the PDAs were reviewed with a nurse practitioner [24] or decision counselor [28].

In Fagan et al. [28] as well intervention arm of Kathuria et al. pilot 2 [24], PDAs were reviewed during a shared decision making encounter with a referral to LCS clinic in Fagan et al. [28]. In three studies, patients participated in a shared decision making encounter with a provider after review of PDA [21, 23, 30]. No shared decision-making encounter was offered after review of PDAs or educational materials in six studies [25–27, 29, 31, 32]. In Niranjan et al. [22], Dharod et al. [33], Volk et al. (both arms) [29] and control arms in Kathuria et al. trials [24] patients were referred to either a primary care provider or LCS clinic after review of PDAs.

Order for LCS was only placed by Ruparek et al. [30] and intervention arm of Kathuria et al. pilot 2 [24]. Further, assistance with LCS scheduling was only conducted in intervention arm of Kathuria et al. pilot 2 [24].

### Quality assessment

In all nine pre-post design studies, clear objectives, eligibility criteria, and outcome measures were addressed (Supplementary Fig. 1). All studies used appropriate statistical measures to analyze outcome changes. One study [26], raised concerns about patient population selection. Additionally, one study [22] did not enroll all eligible patients. Concern about inadequate sample sizes were noted in 7 studies (77.7%) [21–23, 26, 28, 31, 32], with one study [28] reporting over 20% loss to follow-up. Furthermore, one study[26] lacked a clear description and delivery of the intervention. Studies did not clearly define outcome at multiple time points, resulting in unclear bias risk. No studies conducted group-level interventions.

In all four RCTs, no concerns were noted regarding baseline group similarity, drop-out rates, or intervention adherence (Supplementary Fig. 2). Additionally, all studies had prespecified outcomes, used appropriate statistical measures for outcome assessment, and followed an intention-to-treat approach. However, it was unclear in all studies whether arm allocation was concealed. Two studies did not describe blinding for researcher and participants [24, 30], and in one study, participants were not blinded to study arms [29]. Further, one study lacked a sufficiently large sample size to detect a statistically significant difference [24].

### LCS completion rate among patients who received PDA

**Fig. 2:**
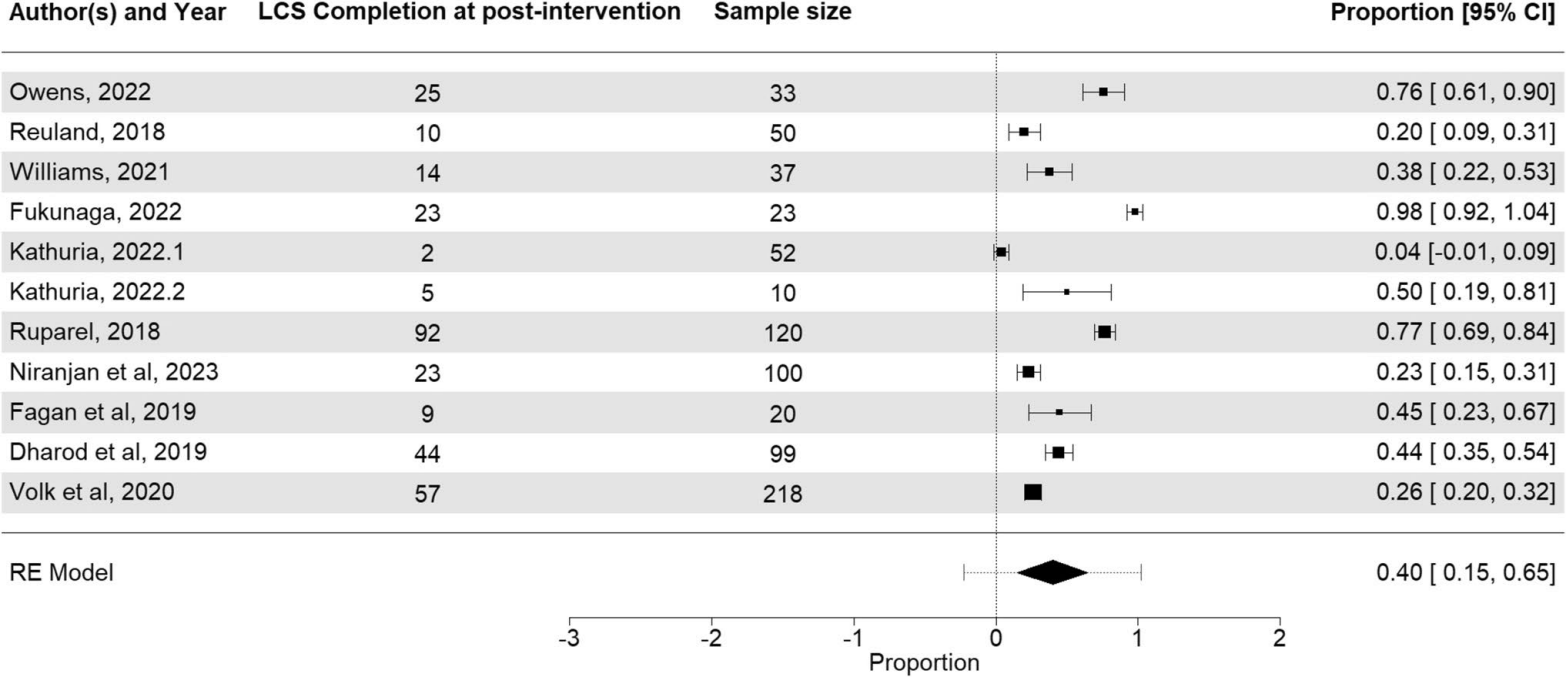
Forest plot of LCS Completion rate among 10 included studies (11 cohorts). The pooled estimate is the outcome of the meta-analysis and is typically explained using a forest plot. The black squares in the forest plot are the Proportion and 95% confidence intervals in each study. The area of the squares represents the weight reflected in the meta-analysis. The black diamond represents the proportion and 95% confidence interval (CI) calculated across all the included studies. (Color figure online)

Overall, 11 trials including 7 pre-post [21–23, 26, 28, 32, 33] and 4 RCTs [24, 29, 30] reported LCS completion rate among patients who received PDA within one to six months after receipt of intervention. Using post-intervention LCS completion data across the eleven cohorts, the pooled estimate for LCS completion rate across all follow-up periods (range, 1–6 months) was 40% (95% confidence interval [CI], 15%, 65%) with significant heterogeneity (I^2^) of 97% (*p* < 0.001) (Fig. 2). There was no significant publication bias (Kendall's tau = 0.20, *p* = 0.44) (Supplementary Fig. 3). Given none of the RCTs had an arm where no PDA or educational material was offered to participants, we could not assess the effectiveness of PDAs' use versus non-use on outcomes.

### Intent to receive LCS among patients who received PDA

**Fig. 3:**
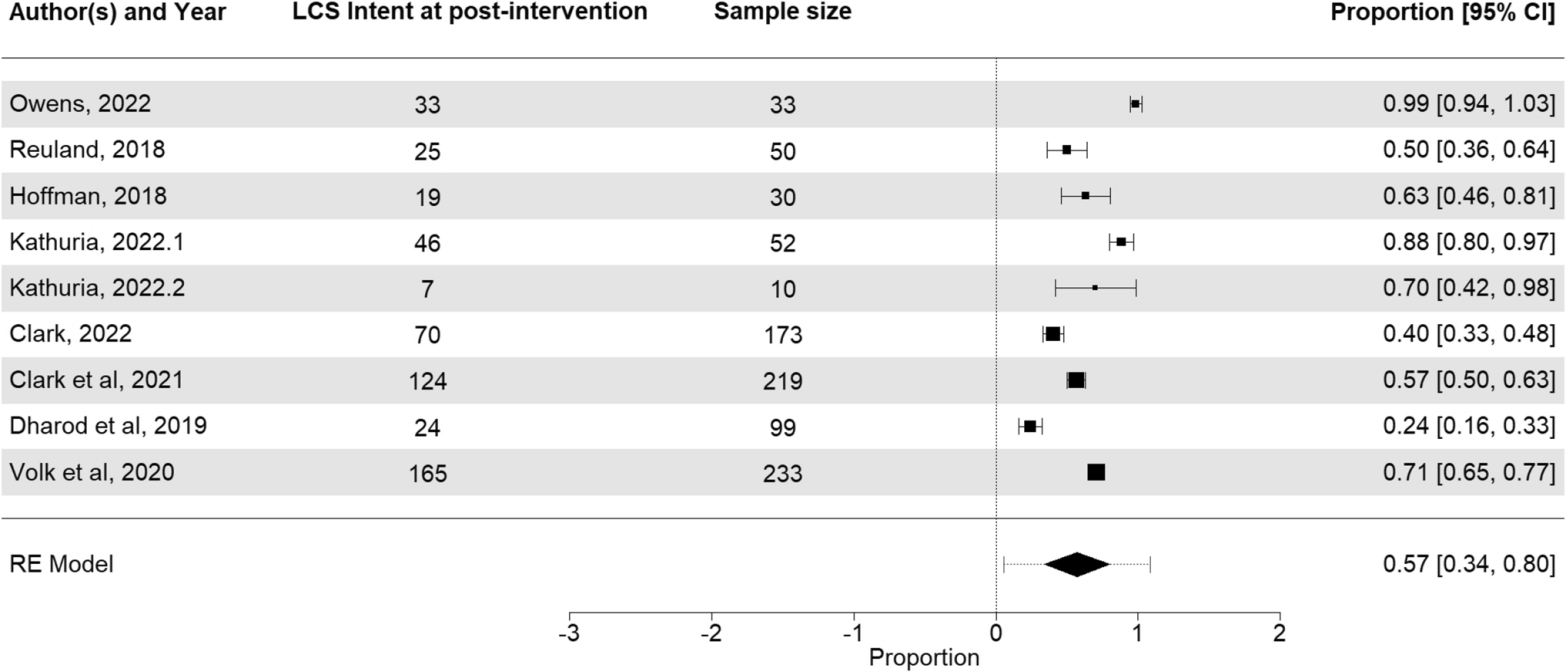
Forest plot of LCS intent among 8 included studies (9 cohorts). The pooled estimate is the outcome of the meta-analysis and is typically explained using a forest plot. The black squares in the forest plot are the Proportion and 95% confidence intervals in each study. The area of the squares represents the weight reflected in the meta-analysis. The black diamond represents the proportion and 95% confidence interval (CI) calculated across all the included studies

Overall, 9 cohorts including 5 pre-post designs [21, 27, 31–33] and 4 RCTs [24, 25, 29] reported intent to receive LCS among patients who received PDA. Using post-intervention intent data across the nine cohorts, the pooled estimate for patient’s intent to complete LCS across all follow-up periods (same day to 3 months) was 57% (95% CI, 34%, 80%) with significant heterogeneity (I^2^) of 96% (*p* < 0.0001) (Fig. 3). There was no significant publication bias (Kendall's tau = − 0.05, *p* = 0.91) (Figure S4). There were not enough observations to calculate changes in intent between pre-post design studies.

## Discussion

In this systematic review and meta-analysis of studies reporting the rate of LCS completion or intent to complete LCS among patients who received PDAs or educational materials, we found that an estimated 40% of patients completed LCS and 57% reported an intention to complete LCS.

A recent study showed that only 42% of patients who received LCS had a documented SDM process in their medical records, and a PDA was used only in 55.6% of documented SDM encounters[10]. Although our study did not assess the impact of PDAs on decisional quality, or conflict, our results demonstrate that LCS completion among patients who receive PDAs is much higher than the current reported utilization rate of 4.5% among Americans [34]. Therefore, it is possible that PDAs can effectively facilitate SDM and play a pivotal role in assisting patients in selecting an option aligned with their values, thereby reducing the percentage of patients who remain undecided or adopt a passive stance in the decision-making process [35]. Furthermore, PDAs contribute to enhancing patient knowledge, alleviating decisional conflict, and fostering improved communication between patients and clinicians [35, 36]. Implementing various forms of PDAs, such as computer-based interfaces, video presentations, informational films, and paper-based material, or a combination of two or more decision aids, provide patients with more interactive ways to gain knowledge about LCS available treatment and care options.

Our study has clinical implications. While CMS mandates the use of decision aids as part of SDM, their utilization in clinical practice remains suboptimal[10]. Our findings support efforts to increase the use of PDAs as part of the SDM for LCS. Strategies for implementing PDAs in clinical practice include training the entire clinical team to understand PDAs intended use and integration into patients' care [37]. Additionally, obtaining buy-in from practice leadership can ensure that team members receive appropriate training to effectively utilize PDAs [37]. Collaborative development of the PDAs by the clinical team allows for the creation of a tool that fits best within their practice and measuring PDA outcomes can showcase the its significance, supporting continuous implementation[37].

Further, pre-identifying eligible patients and standardizing the timely delivery of PDAs, ideally through information technology systems, can further facilitate LCS [38]. Using patient portals to deliver PDAs before visits or providing assistance from team members to complete forms in advance can alleviate time constraints and enhance the overall integration and reach of PDAs.

Our study had several limitations. First, abstracts with unpublished manuscript data were excluded. However, no publication bias was detected in our analysis. Second, due to the initiation of LCS insurance coverage in 2015, there is limited available research on LCS. This led to two constraints: our study has a limited number of RCTs, and all included studies are confined to the last 8 years. Significant heterogeneity was observed among our included studies, and there were differences in the format or modes of delivery of PDAs, which would limit identifying best mode of delivery of PDAs. We could not assess the effectiveness of PDAs on LCS completion or intent, as none of existing RCTs had an arm where PDAs were not offered. Lastly, we did not assess the effectiveness of PDAs on decisional conflict or decisional quality as there were not enough manuscripts using consistent outcome measures to enable a meta-analysis.

In conclusion, we found that LCS completion and intention to complete LCS among patients who use PDAs is high. Our findings support the need to implement PDAs in clinical practice which could further facilitate shared decision-making and improve LCS uptake among eligible patients.

## Supplementary Information

Below is the link to the electronic supplementary material.

Supplementary file1 (DOCX 1136 KB)

## Data Availability

Data generated or analyzed during the study are available from the corresponding author by request.

## Abbreviations

CI: Confidence interval
CINAHL: Cumulative index to nursing and allied health literature
CMS: Centers for medicare & medicaid services
EMR: Electronic medical record
LCS: Lung cancer screening
LDCT: Low dose computed tomography
NHLBI: National Heart, Lung, and Blood Institute
PDA: Patient decision aids
PRISMA: Preferred reporting items for systematic review and meta-analysis
RCT: Randomized controlled trial
USPSTF: US Preventive Services Task Force
