# Supplementary material for: Lung cancer screening completion among patients using decision aids: a systematic review and meta-analysis
Source: Cancer Causes Control. 2025 Mar 18;36(9):871–85. doi: 10.1007/s10552-025-01987-4 (PMC12380944; doi:10.1007/s10552-025-01987-4)

**Lung Cancer Screening Completion Among Patient using Decision Aids: A Systematic Review and Meta-analysis**

**Supplemental Table 1. PubMed Search Strategy**

**Supplemental Table 2. Individual Data Sources Search Strategies.**

**Supplemental Figure 1. Risk of Bias Assessment Pre-Post Studies with The NHLBI Criteria.**

**Supplemental Figure 2. Risk of Bias Assessment of RCT Studies with The NHLBI criteria.**

**Supplemental Figure 3. Funnel plot of LCS Completion rate among 10 included studies (11 cohorts).**

**Supplemental Figure 4. Funnel plot intent for LCS among 8 included studies (9 cohorts).**

**Supplemental Table 1. PubMed Search Strategy**

| Line | Term used |
| --- | --- |
| 1 | "lung neoplasms"[MeSH Terms] |
| 2 | "lung*"[Title] |
| 3 | #1 OR #2 |
| 4 | "Early Detection of Cancer"[MeSH Terms:noexp] |
| 5 | "Mass Screening"[MeSH Terms:noexp] |
| 6 | #4 OR #5 |
| 7 | #3 AND #6 |
| 8 | "lung neoplasms/prevention and control"[MeSH Terms] |
| 9 | "Tomography, x ray computed"[MeSH Terms] |
| 10 | Computed Tomograph*"[Title/Abstract] OR "LDCT"[Title/Abstract] OR "low dose CT"[Title/Abstract] |
| 11 | #8 AND (#9 OR #10) |
| 12 | ("screen*"[Title/Abstract] OR "early detection"[Title/Abstract:~3]) AND "lung"[Title/Abstract] AND ("cancer*"[Title/Abstract] OR "neoplas*"[Title/Abstract] OR "adenocarcinoma*"[Title/Abstract]) |
| 13 | ("screen*"[Title/Abstract] OR "early detection"[Title/Abstract:~3]) AND ("SCLC"[Title/Abstract] OR "NSCLC"[Title/Abstract]) |
| 14 | (Screen* OR (early detect*)) AND (lung*[Title/Abstract] AND (cancer*[Title/Abstract] OR neoplas*[Title/Abstract] OR Adenocarcinoma*[Title/Abstract] OR SCLC[Title/Abstract] OR NSCLC[Title/Abstract])) |
| 15 | (Screen* OR (“early detect*”)) AND (SCLC[Title/Abstract] OR NSCLC[Title/Abstract]) |
| 16 | #12 OR #13 OR #14 OR #15 |
| 17 | #7 OR #11 OR #16 |
| 18 | #17 Filters: English, from 2011 - 3000/12/12 |
| 19 | #18 NOT ("Smoking Cessation"[mh] OR “smoking cessation”) |
| 20 | Case Reports [Publication Type] OR "case series" OR Editorial [Publication Type] OR Comment [Publication Type] OR Letter [Publication Type] OR News [Publication Type] |
| 21 | #19 NOT #20 |
| 22 | "patient compliance"[MeSH Terms] OR ("adher*"[Title/Abstract] OR "complian*"[Title/Abstract] OR "nonadher*"[Title/Abstract] OR "non adher*"[Title/Abstract] OR "comply*"[Title/Abstract] OR "complies"[Title/Abstract] OR "complied"[Title/Abstract] OR "noncomply*"[Title/Abstract] OR "noncomplie*"[Title/Abstract] OR "noncomplian*"[Title/Abstract] OR "non comply*"[Title/Abstract] OR "non complie*"[Title/Abstract] OR "non complian*"[Title/Abstract] OR "overadher*"[Title/Abstract] OR "over adher*"[Title/Abstract] OR "overcomply*"[Title/Abstract] OR "over comply*"[Title/Abstract] OR "overcomplie*"[Title/Abstract] OR "over complie*"[Title/Abstract] OR "overcomplian*"[Title/Abstract] OR "over complian*"[Title/Abstract]) |
| 23 | Patient participation [mh] OR uptake OR awareness OR knowledge OR discussion  Search terms for Interventions |
| 24 | Decision making[mh] OR outreach OR navigation OR patient education OR social media OR Internet OR facebook OR Twitter OR YouTube OR Tiktok OR Instagram OR mobile health OR digital media OR search engine OR community health advisor* OR Decision support OR decision aid OR “Decision Support Techniques”[mh] OR “Communications Media”[mh] OR “Reminder Systems”[mh] OR reminder[tiab] OR “Referral and Consultation”[mh] OR “physician referral” OR provider education OR podcasts |
| 25 | #21 AND (#22 OR #23) AND #24 |
|  | Filters: 2011/01/01:2023/02/01[Date - Publication])) AND 2011/01/01:2023/02/01[Date - Publication])) AND (2011/1/1:2023/2/1[pdat]) |

**Supplemental Table 2. Individual Data Sources Search Strategies.**

| Database | Keywords | Number of publications |
| --- | --- | --- |
| PubMed | "lungneoplasms"[MeSHTerms]"lung*[Title]"EarlyDetectionofCancer[MeSHTerms:noexp]"MassScreening[MeSHTerms:noexp]"lungneoplasms/preentionandcontrol"[MeSHTerms]"Tomography,xraycomputed"[MeSHTerms]"ComputedTomograph*"[Title/Abstract]OR"LDCT"[Title/Abstract]OR"lowdoseCT"[Title/Abstract]("screen[Title/Abstract]OR"earlydetection"[Title/Abstract:~3])AND"lung"[Title/Abstract]AND("cancer*"[Title/Abstract]OR"neoplas*"[Title/Abstract]OR"adenocarcinoma*"[Title/Abstract])("screen*"[Title/Abstract]OR"earlydetection"[Title/Abstract:~3])AND("SCLC"[Title/Abstract]OR "NSCLC"[Title/Abstract])(Screen* OR (early detect*)) AND (lung*[Title/Abstract] AND(cancer*[Title/Abstract]ORneoplas*[Title/Abstract] ORAdenocarcinoma[Title/Abstract]ORSCLC[Title/Abstract]OR NSCLC[Title/Abstract]))(Screen* OR (“early detect*”)) AND (SCLC[Title/Abstract] OR NSCLC[Title/Abstract]Filters: English, from 2011 - 3000/12/12 NOT ("Smoking Cessation"[mh] OR “smoking cessation”)Case Reports [Publication Type]OR"caseseries"OREditorial[PublicationType]ORComment[PublicationType]ORLetter[PublicationType]ORNews[PublicationType]"patientcompliance"[MeSHTerms]OR("adher*"[Title/Abstract]OR"complian*"[Title/Abstract]OR"nonadher*"[Title/Abstract]OR"nonadher*"[Title/Abstract]OR"comply*"[Title/Abstract]OR"complies"[Title/Abstract]OR"complied"[Title/Abstract]OR"noncomply*"[Title/Abstract]OR"noncomplie*"[Title/Abstract]OR"noncomplian*"[Title/Abstract]OR"noncomply*"[Title/Abstract]OR"noncomplie*"[Title/Abstract]OR"noncomplian*"[Title/Abstract]OR"overadher*"[Title/Abstract]OR"overadher*"[Title/Abstract]OR"overcomply*"[Title/Abstract]OR"overcomply*"[Title/Abstract]OR"overcomplie*"[Title/Abstract]OR"overcomplie*"[Title/Abstract]OR"overcomplian*"[Title/Abstract]OR"over complian*"[Title/Abstract]) Patient participation [mh] OR uptake OR awareness OR knowledge OR discussion Decision making[mh] OR outreach OR navigation OR patient education OR social media OR Internet OR facebook OR Twitter OR YouTube OR Tiktok OR Instagram OR mobile health OR digital media OR search engine OR communityhealthadvisor* ORDecisionsupportORdecisionaid OR“DecisionSupportTechniques”[mh]OR“CommunicationsMedia”[mh]OR“ReminderSystems”[mh]ORreminder[tiab]OR“ReferralandConsultation”[mh]OR“physicianreferral”ORprovidereducationORpodcastsFilters:2011/01/01:2023/02/01[Date-Publication]))AND2011/01/01:2023/02/01[Date-Publication]))AND (2011/1/1:2023/2/1[pdat]) | 514 |
| Scopus | ( TITLE-ABS-KEY ( "Lung neoplasm*"  OR  "Lung Cancer"  OR  "Lung cancerprevention" )  AND  TITLE-ABS-KEY ( "Early detection"  OR  "massscreening"  OR  screen* )  AND  TITLE-ABS-KEY ( "patientcomplian*"  OR  nonadheren*  OR  "nonadheren*"  OR  complied  OR  comply  OR  noncomplian*  OR  "noncomplian*"  OR  adheren*  OR  overcomplian*  OR  "overcomplian*"  OR  "Patient participation"  OR  uptake  OR  awareness  OR  knowledge  OR  discussion )  AND  TITLE-ABSKEY ( "Decisionmaking"  OR  outreach  OR  navigat*  OR  "patient education"  OR  "social media"  OR  internet  OR  facebook  OR  twitter  OR  youtube  OR  tiktok  OR  instagram  OR  "mobilehealth"  OR  "smartphone*"  OR  "search engine"  OR  "communityhealth advisor*"  OR  "Decision support"  OR  "decisionaid"  OR  media  OR  reminder*  OR  referral  OR  consultation  OR  "provider education"  OR  podcast* )  ANDNOT  TITLE-ABS-KEY ( "smokingcessation"  OR  "spinaltumor*" ) )  AND  PUBYEAR  >  1987  AND  PUBYEAR  <  2024  AND  ( LIMITTO ( DOCTYPE ,  "ar" )  OR  LIMIT-TO ( DOCTYPE ,  "re" )  OR  LIMIT-TO ( DOCTYPE ,  "cp" ) )  AND  ( EXCLUDE ( EXACTKEYWORD ,  "Breast Cancer" )  OR  EXCLUDE ( EXACTKEYWORD ,  "Cancer Patient" )  OR  EXCLUDE ( EXACTKEYWORD ,  "Prostate Cancer" )  OR  EXCLUDE ( EXACTKEYWORD ,  "Uterine Cervix Cancer" )  OR  EXCLUDE ( EXACTKEYWORD ,  "Animals" ) )  AND  ( EXCLUDE ( SRCTYPE ,  "k" ) ) | 202 |
| Web of Science | ("Lung neoplasm*" OR "Lung Cancer" OR Lung cancer prevention) AND ("Early detect*" OR "mass screening" OR screen* OR early diagno*) (All Fields) and "patient complian*" OR nonadheren* OR "non-adheren*" OR complied OR comply OR noncomplian* OR "non-complian*" OR adheren* OR overcomplian* OR "over complian*" OR "Patient participation" OR uptake OR awareness OR knowledge OR discussion (All Fields) and "Decision making" OR outreach OR navigat* OR "patient education" OR "social media" OR Internet OR Facebook OR Twitter OR YouTube OR TikTok OR Instagram OR "mobile health" OR "smartphone*" OR "search engine" OR "community health advisor*" OR "Decision support" OR "decision aid" OR Media OR Reminder* OR Referral OR Consultation OR "provider education" OR podcast* (All Fields) not "smoking cessation" OR "breast cancer" OR "prostate cancer" OR "Uterine Cervix Cancer" (All Fields) and Letter or Book Chapters or Editorial Material (Exclude – Document Types) | 391 |
| Cochrane Central | ("Lung neoplasm*" OR "Lung Cancer" OR Lung cancer prevention) AND ("Early detection" OR "mass screening" OR screen* OR early diagno*) in Title Abstract Keyword AND "patient complian*" OR nonadheren* OR "non-adheren*" OR complied OR comply OR noncomplian* OR "non-complian*" OR adheren* OR overcomplian* OR "over complian*" OR "Patient participation" OR uptake OR awareness OR knowledge OR discussion in Title Abstract Keyword AND "Decision making" OR outreach OR navigat* OR "patient education" OR "social media" OR Internet OR Facebook OR Twitter OR YouTube OR TikTok OR Instagram OR "mobile health" OR "smartphone*" OR "search engine" OR "community health advisor*" OR "Decision support" OR "decision aid" OR Media OR Reminder* OR Referral OR Consultation OR "provider education" OR podcast* in Title Abstract Keyword NOT "smoking cessation" OR "breast cancer" OR "prostate cancer" OR "Uterine Cervix Cancer" in Title Abstract Keyword - (Word variations have been searched) | 70 |
| EBSCO CINAHL | TX ( (“Lung neoplasm*" OR "Lung Cancer" OR "Lung cancer prevention") AND ("Early detect*" OR "mass screening" OR screen* OR “early diagnos*") ) AND ( "patient complian*" OR nonadheren* OR "non-adheren*" OR complied OR comply OR noncomplian* OR "non-complian*" OR adheren* OR overcomplian* OR "over complian*" OR "Patient participation" OR uptake OR awareness OR knowledge OR discussion ) AND ( "Decision making" OR outreach OR navigat* OR "patient education" OR "social media" OR Internet OR Facebook OR Twitter OR YouTube OR TikTok OR Instagram OR "mobile health" OR "smartphone*" OR "search engine" OR "community health advisor*" OR "Decision support" OR "decision aid" OR Media OR Reminder* OR Referral OR Consultation OR "provider education" OR podcast* ) NOT ( "smoking cessation" OR "breast cancer" OR "prostate cancer" OR "Uterine Cervix Cancer" ) | 102 |

**Supplemental Figure 1. Risk of Bias Assessment Pre-Post Studies with The NHLBI Criteria.** NHLBI Risk of Bias criteria for pre-post studies(40) are observed through graph (a) and a summary (b). Review authors’ judgments about each domain presented as percentages across included studies.

**A.**

**
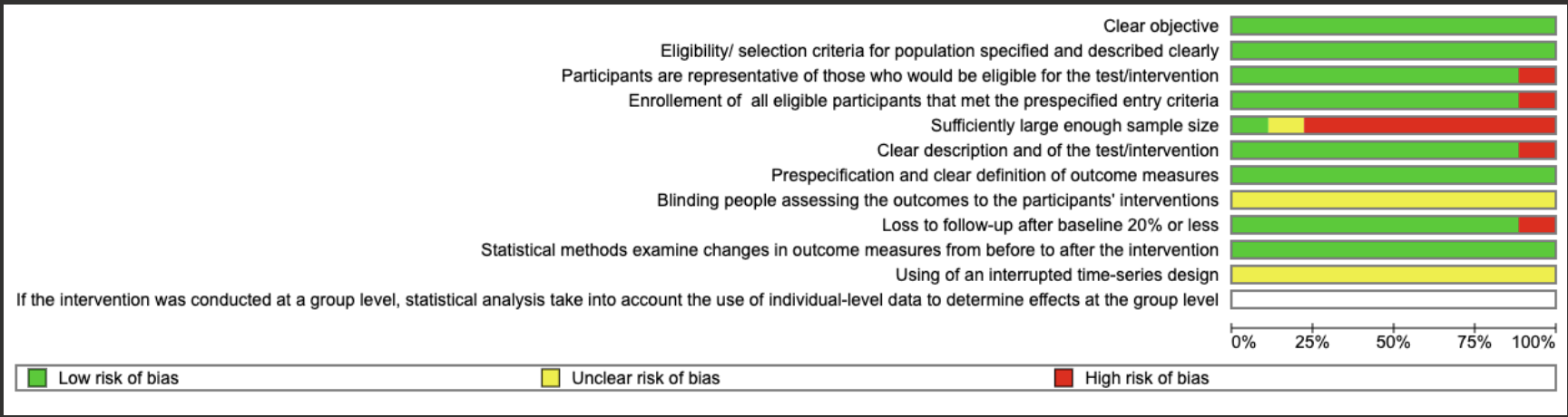
**

**B.**


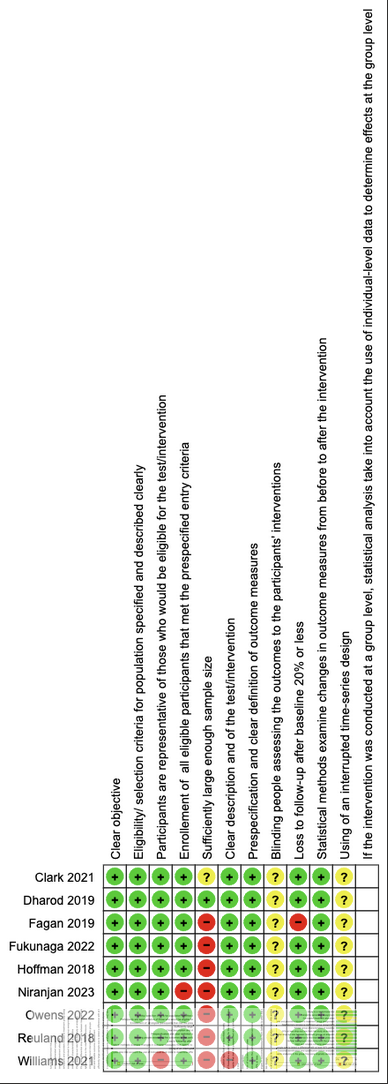
**Supplemental Figure 2. Risk of Bias Assessment of RCT Studies with The NHLBI Criteria.** NHLBI Risk of Bias criteria for randomized control trials (40) is observed through graph (a) and a summary (b).

**A.**

**
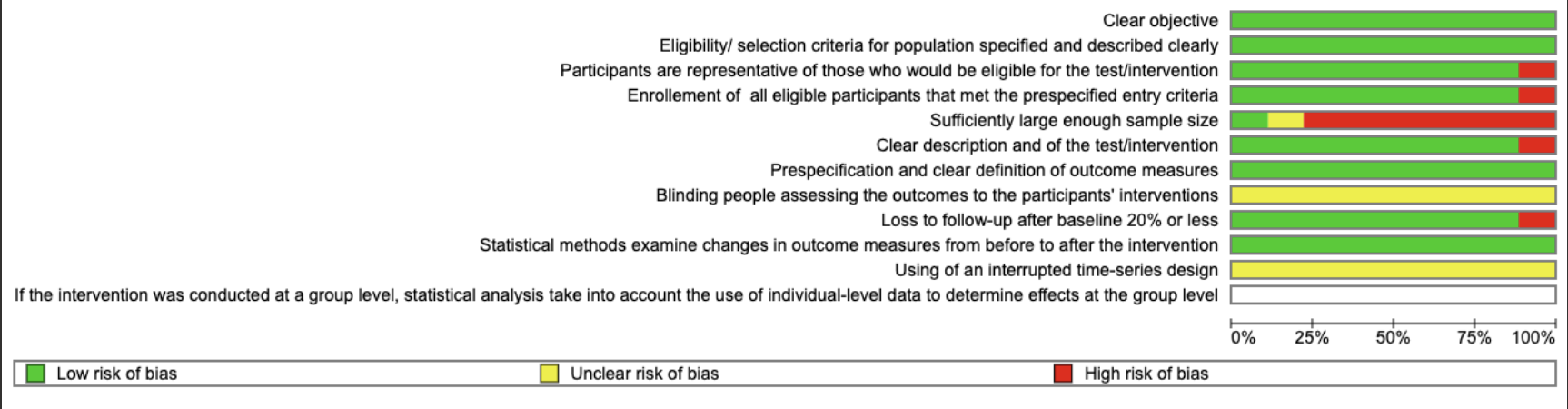
**

**B.**

**
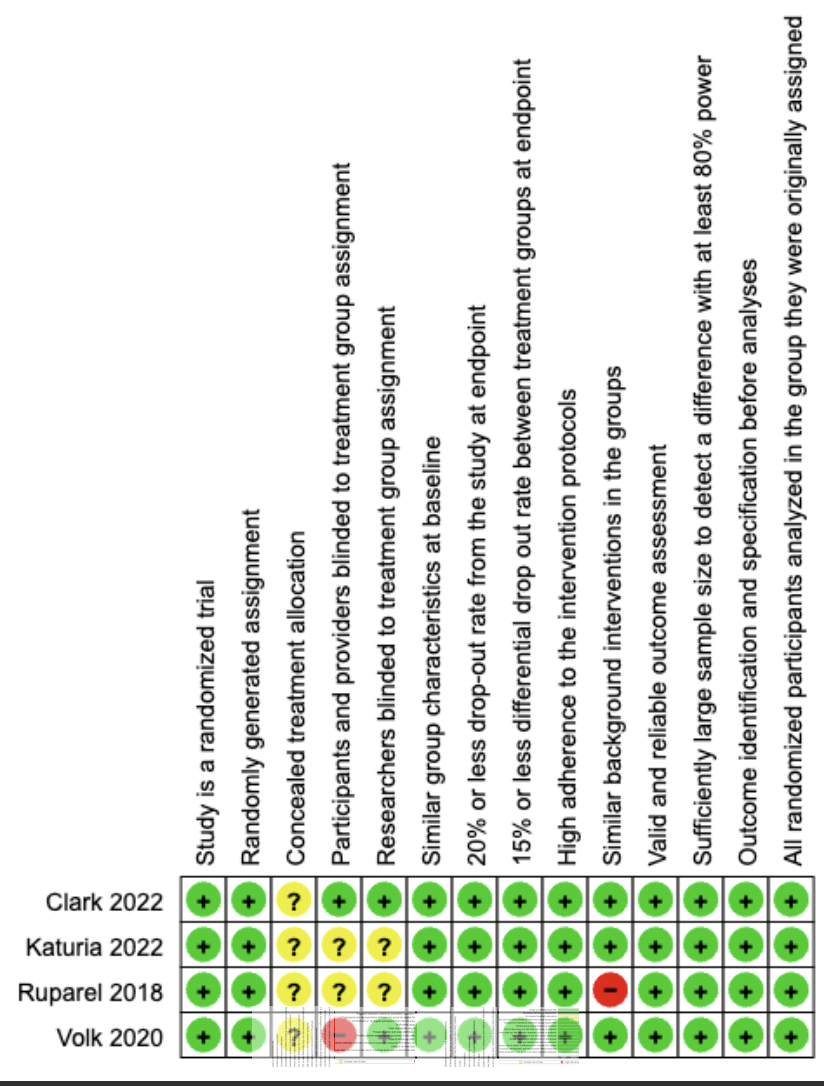
**

**Supplemental Figure 3. Funnel plot of LCS Completion rate among 10 included studies (11 cohorts).** For random-effects models (i.e., models not involving moderators), the plot shows the observed effect sizes or outcomes on the x-axis against the corresponding standard errors on the y-axis. A vertical line indicates the estimate based on the model. A pseudo confidence interval region is drawn around this value with bounds equal to ±1.96SE, where SE is the standard error value from the y-axis (assuming level=95).


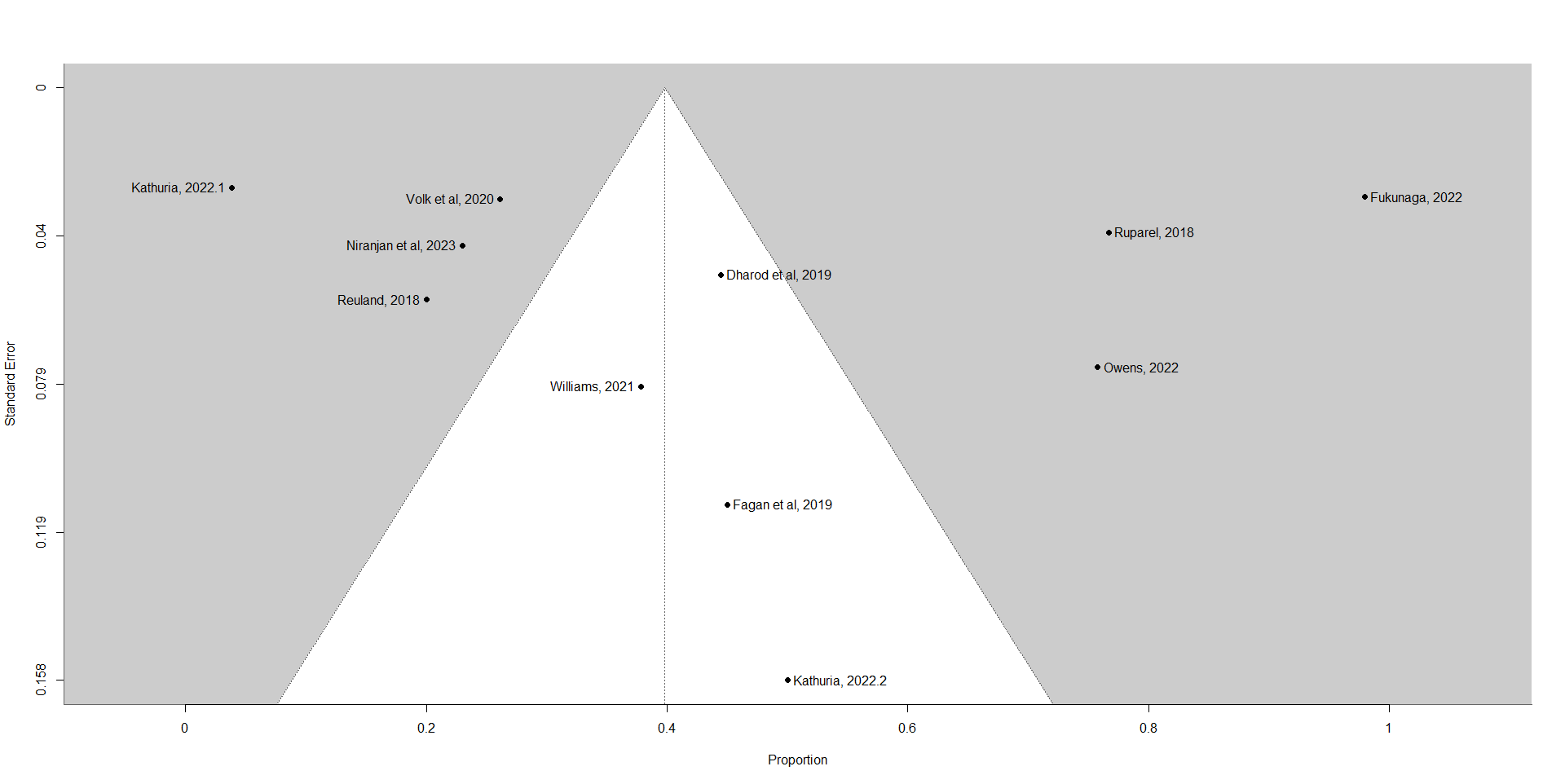


**Supplemental Figure 4. Funnel plot intent for LCS among 8 included studies (9 cohorts).**  For random-effects models (i.e., models not involving moderators), the plot shows the observed effect sizes or outcomes on the x-axis against the corresponding standard errors on the y-axis. A vertical line indicates the estimate based on the model. A pseudo confidence interval region is drawn around this value with bounds equal to ±1.96SE, where SE is the standard error value from the y-axis (assuming level=95).


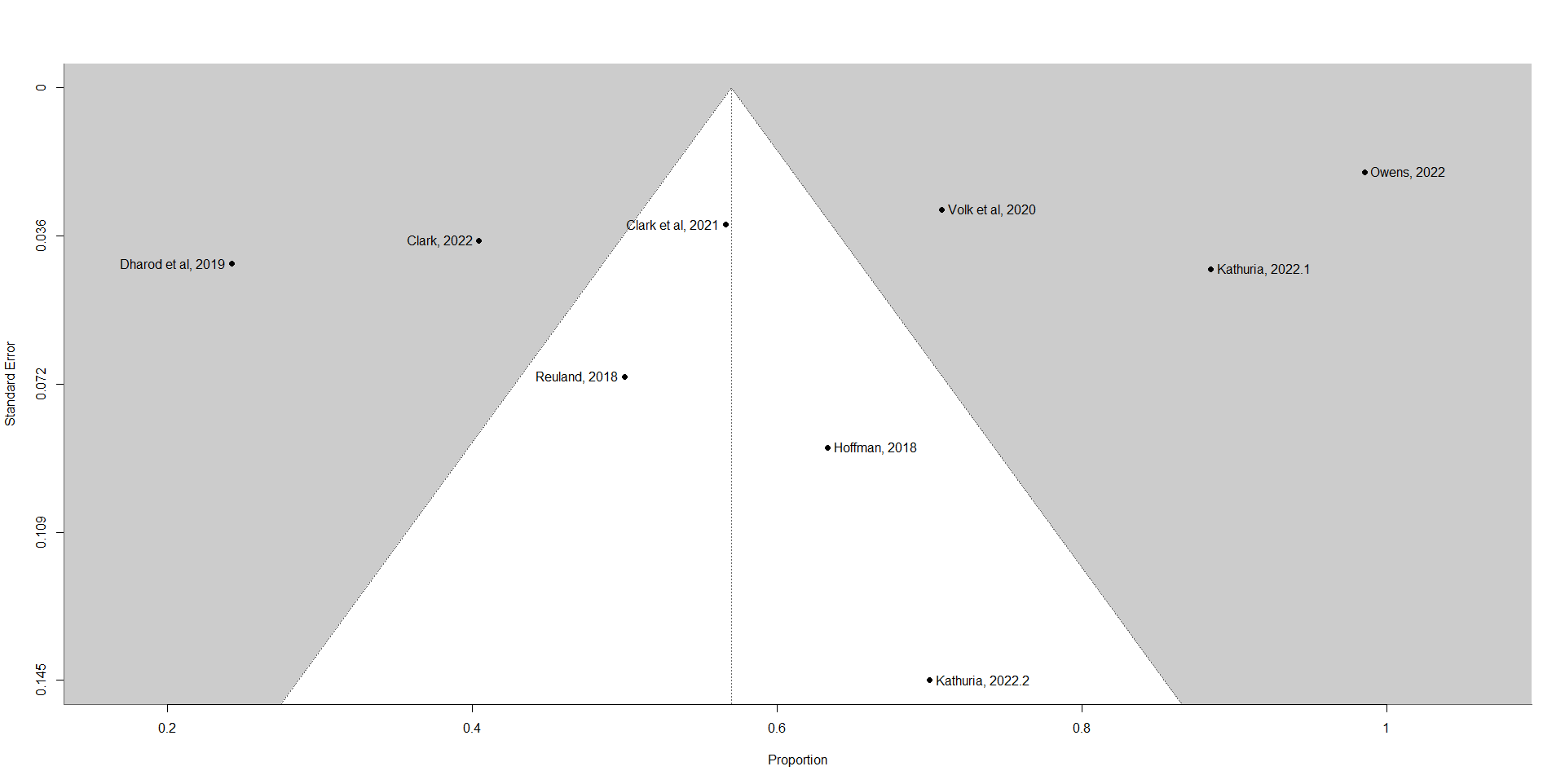

Supplement: Supplementary file 1 — Supplementary file1 (DOCX 1136 KB) [file 10552_2025_1987_MOESM1_ESM.docx]
